# Bridging Hypertension Care Shortfalls Between Provider Capacity and Patient Needs: A Pooled Analysis of Data From 199 Countries and Territories

**DOI:** 10.1161/HYPERTENSIONAHA.125.24783

**Published:** 2025-09-26

**Authors:** Shiva Raj Mishra, Gautam Satheesh, Vishnu Khanal, Bipin Adhikari, Daniel Parker, Dean S. Picone, Niamh Chapman, Aletta E. Schutte, Richard I. Lindley

**Affiliations:** Nepal Development Society, Bharatpur, Chitwan (S.R.M., V.K.).; School of Health Sciences (G.S., D.S.P., N.C.), The University of Sydney, New South Wales, Australia.; Westmead Applied Research Center (R.I.L.), The University of Sydney, New South Wales, Australia.; Remote Health Systems and Climate Change Centre, Menzies School of Health Research, Charles Darwin University, Alice Springs, Northern Territory, Australia (V.K.).; Mahidol-Oxford Tropical Medicine Research Unit, Faculty of Tropical Medicine, Mahidol University, Bangkok, Thailand (B.A.).; Department of Population Health and Disease Prevention, University of California Irvine (D.P.).; School of Population Health, The University of New South Wales, Australia (A.E.S.).; The George Institute for Global Health, Sydney, New South Wales, Australia (A.E.S.).

**Keywords:** blood pressure, community health workers, hypertension, pharmacists, physicians

## Abstract

**BACKGROUND:**

This study estimates the overall gaps between health system capacity of physician and nonphysician providers (nurses, pharmacists, and community health workers) and patients’ needs for hypertension management across country income groups.

**METHODS:**

We extracted data on population, physician, and nonphysician density (nurses, pharmacists, community health workers) per 10 000 people from the World Bank Databases for 199 countries in 2021. Data on hypertension prevalence were obtained from the Non-Communicable Disease Risk Factor Collaboration (NCD-RisC) in 2021. We estimated patient need for clinic visits under 4 scenarios: 12 visits per patient per year (high demand [base scenario, reflecting common practice in many low- and middle-income countries, where physicians conduct monthly visits for medication refills]), 6 visits (intermediate scenario), 3 (low scenario), 1 (minimal scenario) and 2 scenarios based on health worker capacity to provide clinic services: 20 patients per day (base capacity) and 10 patients per day (low capacity) per provider.

**RESULTS:**

The overall prevalence of hypertension was 37.5 (SD, 6.6%): 36.2 (7.0%) in high-income countries, 40.3 (6.7) in upper middle–income countries, 36.1 (5.7%) in lower middle–income countries, and 36.7(4.8%) in low-income countries. Physicians (mean±SD, 19.2±17.4), nurses (47.3±54.1), pharmacists (3.9±4.7) per 10 000 were higher in high-income countries, whereas community health workers (3.4±7.3) were higher in low- and middle-income countries. All countries showed workforce deficits in the high-demand scenario, which eased under intermediate and minimal scenarios. Incorporating team-based care further reduced these deficits, yielding net surpluses in 36 countries.

**CONCLUSIONS:**

Our analysis highlights significant global health service capacity gaps if hypertension management continues to rely solely on physicians. Addressing these gaps requires expanding team-based care, improving training, and enhancing healthcare infrastructure.

**NOVELTY AND RELEVANCE**

**What Is New?**

This is the first global study quantifying the gap between health system workforce capacity and the population need for hypertension management across 199 countries.
It models care needs under different scenarios of visit frequency (1–12 visits per year) and provider capacity (10–20 patients per day), considering 3 models of care: physician-only, nonphysician-only, and team-based care.
It highlights how including team-based care significantly reduces service delivery gaps.

**What Is Relevant?**

Most low- and middle-income countries face major service shortfalls when relying solely on physicians, even under minimal care assumptions.
Incorporating team-based care (nurses, pharmacists, and community health workers) enables 36 countries to meet or exceed care demand.
Findings support global strategies like the WHO’s HEARTS package promoting team-based, primary care–oriented hypertension services.

**Clinical/Pathophysiological Implications?**

Gaps in care capacity directly contribute to suboptimal blood pressure control and increased cardiovascular disease risk.
Team-based care through task-sharing can enhance diagnosis, medication adherence, and long-term hypertension control outcomes.
Reconfiguring workforce models is essential to close equity gaps and meet global non-communicable disease targets, supporting future implementation of WHO’s HEARTS package.

More than 1.3 billion adults are estimated to have high blood pressure (BP) globally, which leads to ≈11 million deaths per year.^1–3^ The absolute number of people living with hypertension has nearly doubled in the past 30 years, with 90% of growth seen in low- and middle-income countries (LMICs).^2^ There are significant disparities in BP control globally, with high-income countries (HICs) having roughly double the hypertension awareness (71% versus 39%) and treatment (52% compared with 28%), and four times the control rates of LMICs (37% versus 10%).^2,4,5^ Despite advances in BP measurement technology and the availability of safe and effective antihypertensive medicines, a large proportion of patients with hypertension are undetected and do not achieve BP control.^6^

Availability of qualified health professional human resources is a central element for increasing access to quality hypertension services.^7^ In many countries, hypertension services are often provided by nonphysician health workers, such as nurses, or allied health professionals with limited training on hypertension due to shortages of physicians.^8^ Hypertension professionals who are either a physician or a trained nonphysician health worker are needed for long-term follow-up to monitor progress and refill medications. However, the availability of hypertension professionals remains scarce for a large proportion of the global population. The migration of qualified health workers from low-income countries (LICs) and lower middle–income countries to higher-income countries further complicates the situation.^9^ Thus, for addressing the gaps in hypertension care, adoption of team-based, protocol-driven approaches to hypertension management have received interest globally.^8,10,11^ Evidence suggests that multidisciplinary care models—incorporating nonphysician health workers, such as nurses, pharmacists, and community health workers—enhance treatment adherence, patient monitoring, and overall BP management outcomes.^8,11–14^

Despite the literature assessing gaps in hypertension services globally,^3,15^ no studies have systematically estimated the global gap between health system capacity (ability to provide clinic visits by physician or nonphysician health workers, such as pharmacists, nurses, and community health workers) and patient need (number of clinic visits needed to treat and monitor hypertension based on prevalence). The objective of this study was to estimate the overall capacity of health systems to meet demand for management of patients with hypertension, both globally and across different income regions.

## Methods

### Data Availability

Data used in this study is available freely from GitHub repository https://github.com/shivarajmishra/htnglobalanalysis

### Data Sources

We extracted data on the country’s population, physicians, and nonphysician providers (nurses, pharmacists, and community health workers) density from the World Bank Databases.^16^ Data on the prevalence of hypertension were obtained from the Non-Communicable Disease Risk Factor Collaboration (NCD-RisC) 2021 (https://www.ncdrisc.org/).^17^ The NCD-RisC provides data on estimated prevalence of hypertension by country (hypertension defined as systolic BP ≥140/90 mm Hg, diastolic BP ≥90 mm Hg, or taking medication of hypertension).^3^ Countries with nonmissing data on physician density were included in the analysis; yielding 199 countries and territories classified as: HIC (n=61), upper middle income (UMIC; n=64), lower middle income countries (L-MIC) (n=49) and LIC (n=25) in the final analysis based on the World Bank income classification 2023.^18^ We combined physician density with available nonphysician density for each country to calculate total health provider density for team-based care. Consistent with previous studies, our definition of community health workers includes: paraprofessionals or lay individuals who live in the community they serve and have received a brief, standardized job-specific training of short duration, aiming to deliver culturally appropriate health services.^18–20^ For community health workers density per 10 000, data were only available from 96 countries. All data and materials have been made publicly available online at https://github.com/shivarajmishra/htnglobalanalysis.

### Estimation Method for Health System Capacity and Demand for Management of Patients With Hypertension

We estimated the demand for clinic visits for hypertension care by multiplying total number of patients with hypertension by the number of clinical visits per year per patient with hypertension, expanding on methodological work from previous studies.^21,22^ We explored 4 scenarios for clinic visit demand for all patients with hypertension: 12 visits per year (base high-demand scenario), 6 visits per year (intermediate), 3 visits per year per patient (low), and 1 visit (minimal scenario), for physicians only, nonphysicians only, and team-based care (physicians and nonphysicians combined). Maintaining a regular schedule for health care visits allows better monitoring of medication and possible side effects, as well as identifying early signs of worsening symptoms.^23^ International committees such as the American Heart Association, and National Institute for Health and Care Excellence, and the Joint National Committee on Detection, Evaluation, and Treatment of High Blood Pressure recommend regular follow-up on monthly intervals until BP is stable and controlled, and recommends more frequent visits may be needed for those with stage 2 hypertension and have comorbid conditions.^24–26^ Although the health service utilization pattern can vary widely across countries, our scenarios reflect low-income settings, where long-term follow-up is typically undertaken monthly due to medication dispensing practices.

Under the base scenario, we assumed a medical practitioner such as a physician or a nonphysician health worker provides services to either: 20 patients per day (base scenario, that is, 4000 visits per year=20 patients per day×200 days per year), or 10 patients per day (low-capacity scenario, that is, 2000 visits per year), with a total of 200 workdays per year. We applied a base scenario of 20 patients per day, comparable to previous studies^21,22^; however, we used a lower capacity scenario of 10 patients per day, given the diversity of the workforce and their capacity in providing hypertension services across diverse settings. For physicians and nonphysician health workers, we assumed that they spent, on average, 10% of their time on providing hypertension services, consistent with the prior studies.^21,22^ We incorporated 95% uncertainty intervals in hypertension care gaps by using hypertension prevalence estimates and their corresponding 95% CIs provided by the NCD-RisC collaboration.^17^

### Country Classification

Our country classification is based on the World Bank classification: LIC were defined as countries with gross national income of US$ ≤1085, L-MIC (US$1086–US$4255), UMIC (US$4255– US$13 845), and HIC (US$ ≥13 845).^27^

## Results

### Prevalence of Hypertension and Capacity of the Health System

**Figure 1.**
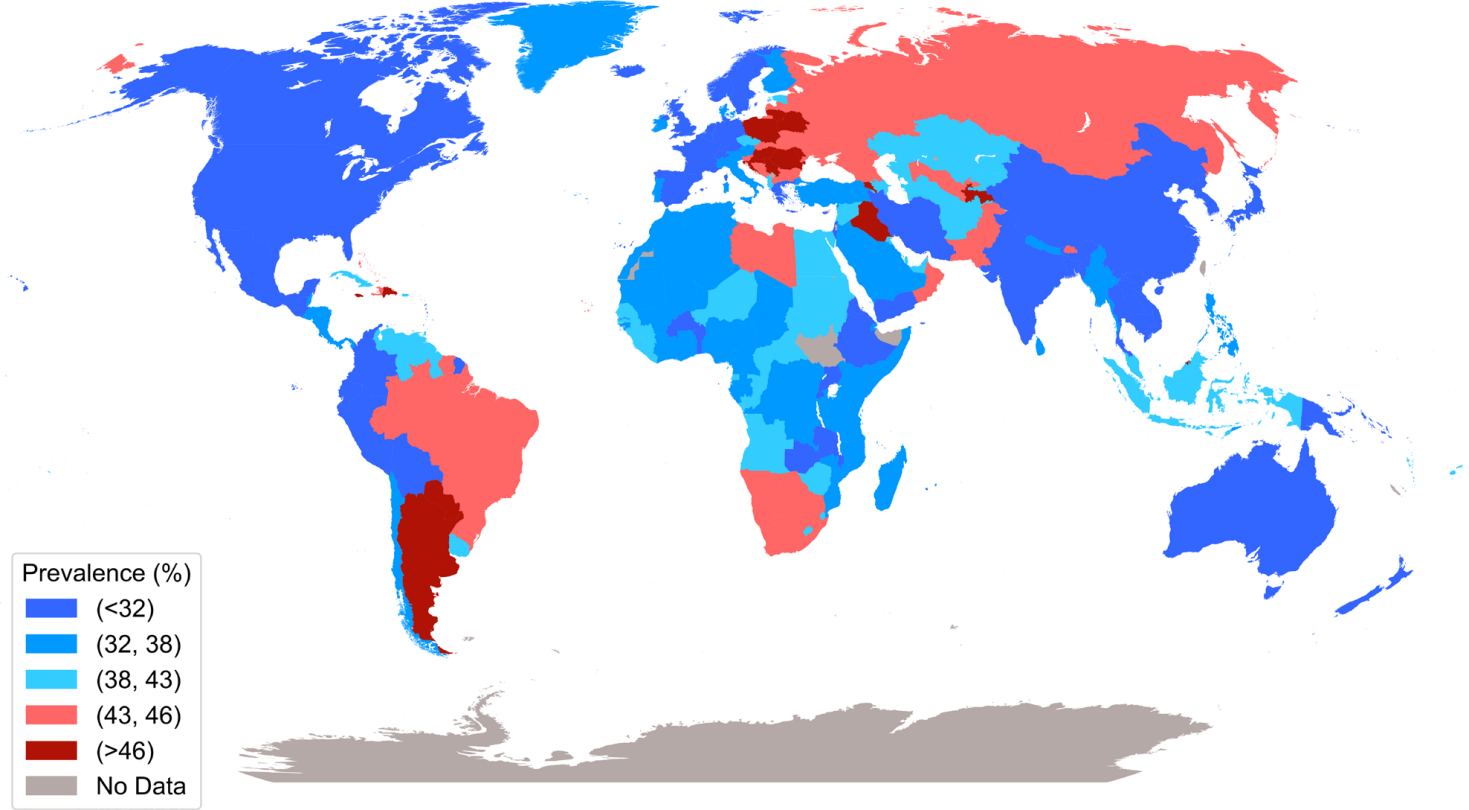
**Prevalence of hypertension globally 2021 (source: Non-Communicable Disease Risk Factor Collaboration [NCD-RisC] Collaboration** https://www.ncdrisc.org/**).**

The 199 countries have a combined population of 8.0 billion people, of which 1.8 billion are estimated to have hypertension. The overall prevalence of hypertension was 37.5 (SD, 6.6%): 36.2 (7.0%) in HIC, 40.3 (6.7) in UMIC, 36.1 (5.7%) in L-MIC, and 36.7(4.8%) in LIC (Figure 1).

**Figure 2.**
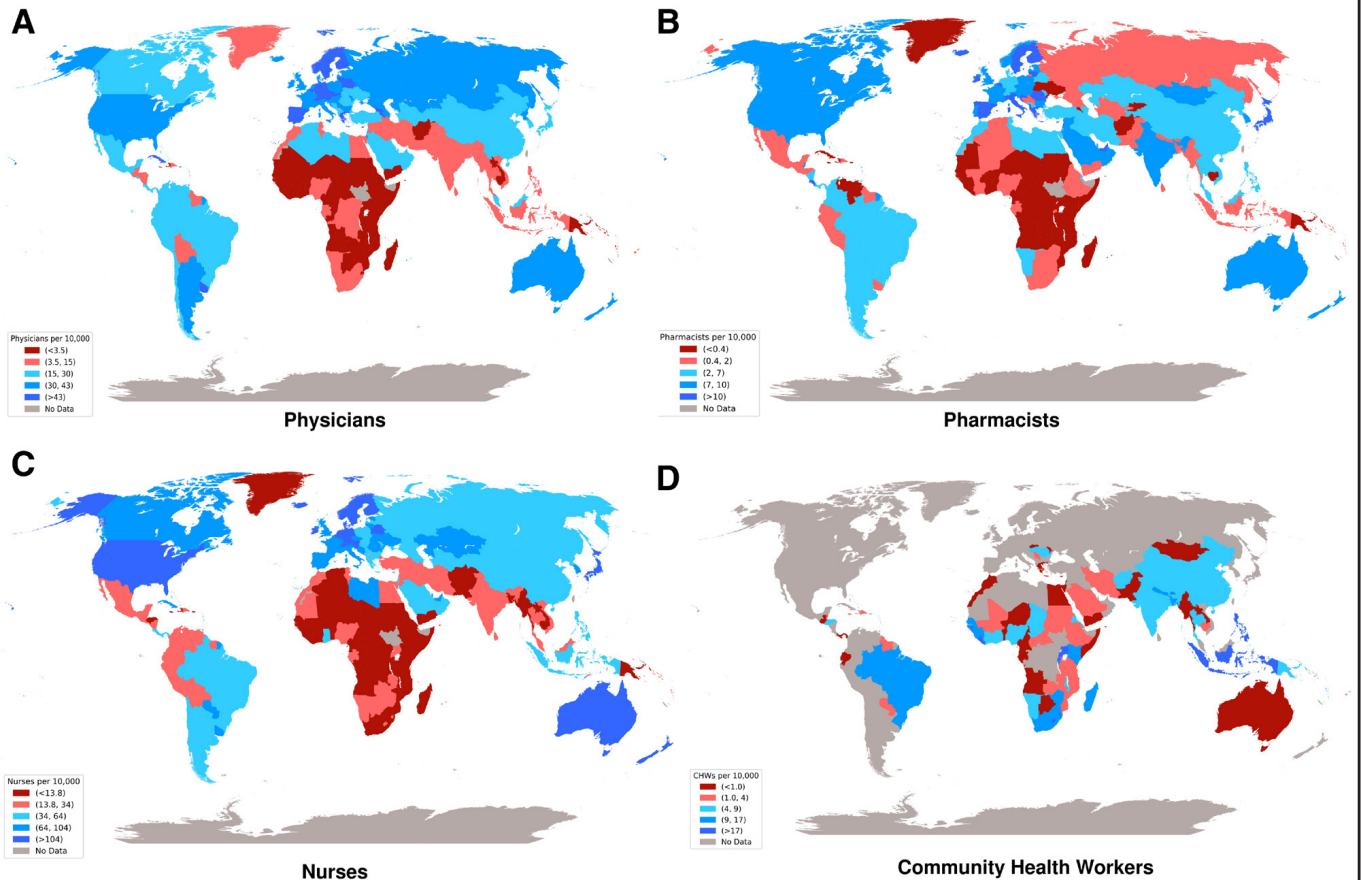
**Density of various providers across regions.** Physician density (**A**), pharmacist density (**B**), nurse density (**C**), and community health workers (**D**) per 10 000 population.

**Figure 3.**
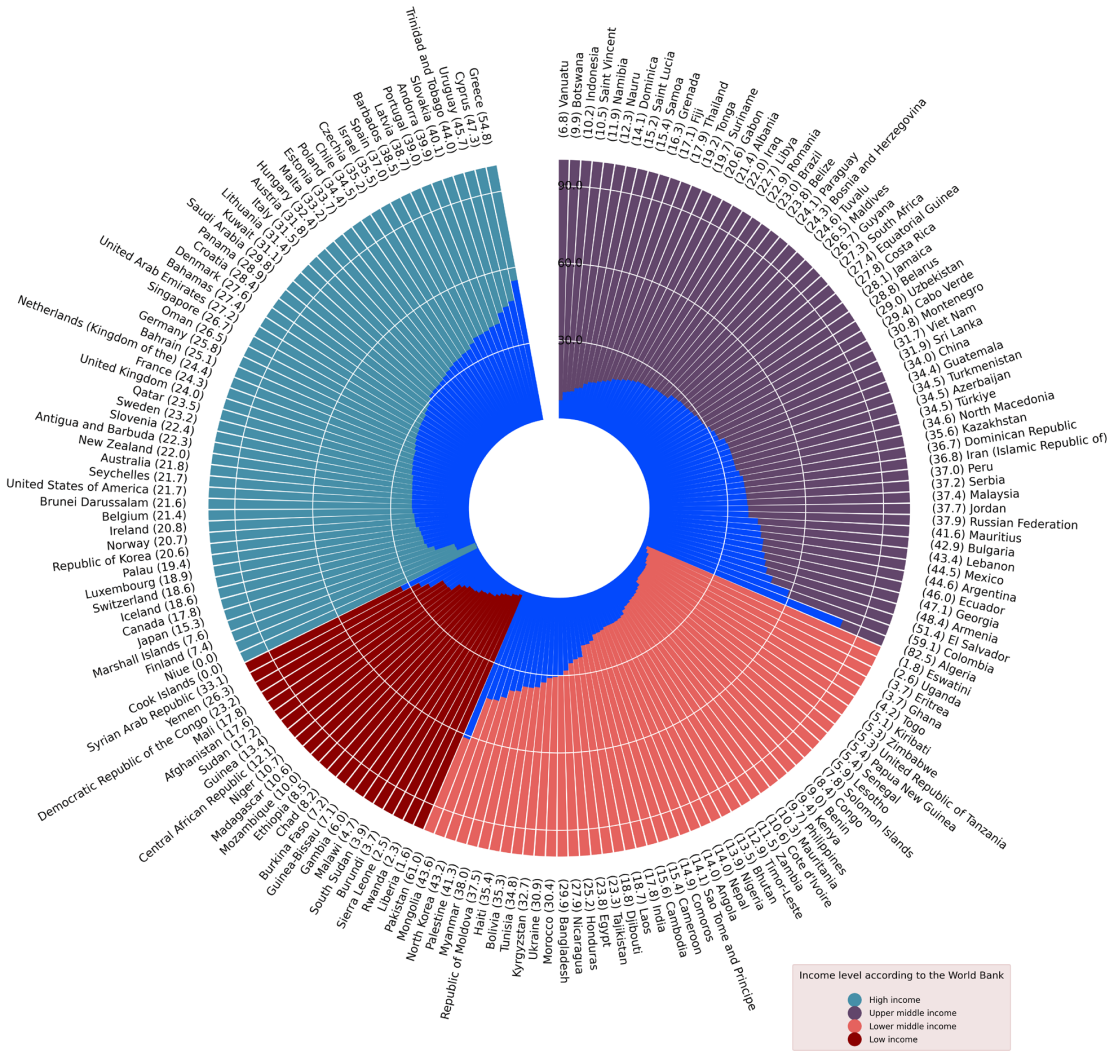
**Clinical visits contributed by physicians (%) and nonphysician health workers (%) across World Bank income regions.** Graph shows the percentage of physicians in total workforce (blue bar), and corresponding text label (country [%]).

Figure 2A shows physician density (per 10 000) by countries. The darkest red shows countries with lowest density. Overall, physician density per 10 000 population varied substantially based on income regions: 1.9 per 10 000 in LIC, 7.8 per 10 000 in L-MIC, 20.7 per 10 000 in UMIC, and 33.8 per 10 000 in HIC. Nurse density per 10 000 (14.8 in LIC, 20.8 in L-MIC, 40.8 in UMIC, and 88.7 in HIC) and pharmacists per 10 000 (0.6 per 10 000 in LIC, 1.7 per 10 000 in L-MIC, 3.1 per 10 000 in UMIC, and 8.0 per 10 000 in HIC) were notably higher across income regions. For community health workers density per 10 000, data were only available from 96 countries, showing variation across regions: highest of 44.9 per 10 000 in Swaziland and lowest of <1 per 10 000 in Angola. Overall, the density of all health workers combined increased progressively with increasing wealth: 20.1 per 10 000 in LIC, 36.7 per 10 000 in L-MIC, 67.1 per 10 000 in UMIC, and 131.0 per 10 000 in HIC. Figure 3 shows the proportion of clinical visits by physicians and non-physicians across regions.

### Estimation of Clinical Visit Deficits by Physicians

**Figure 4.**
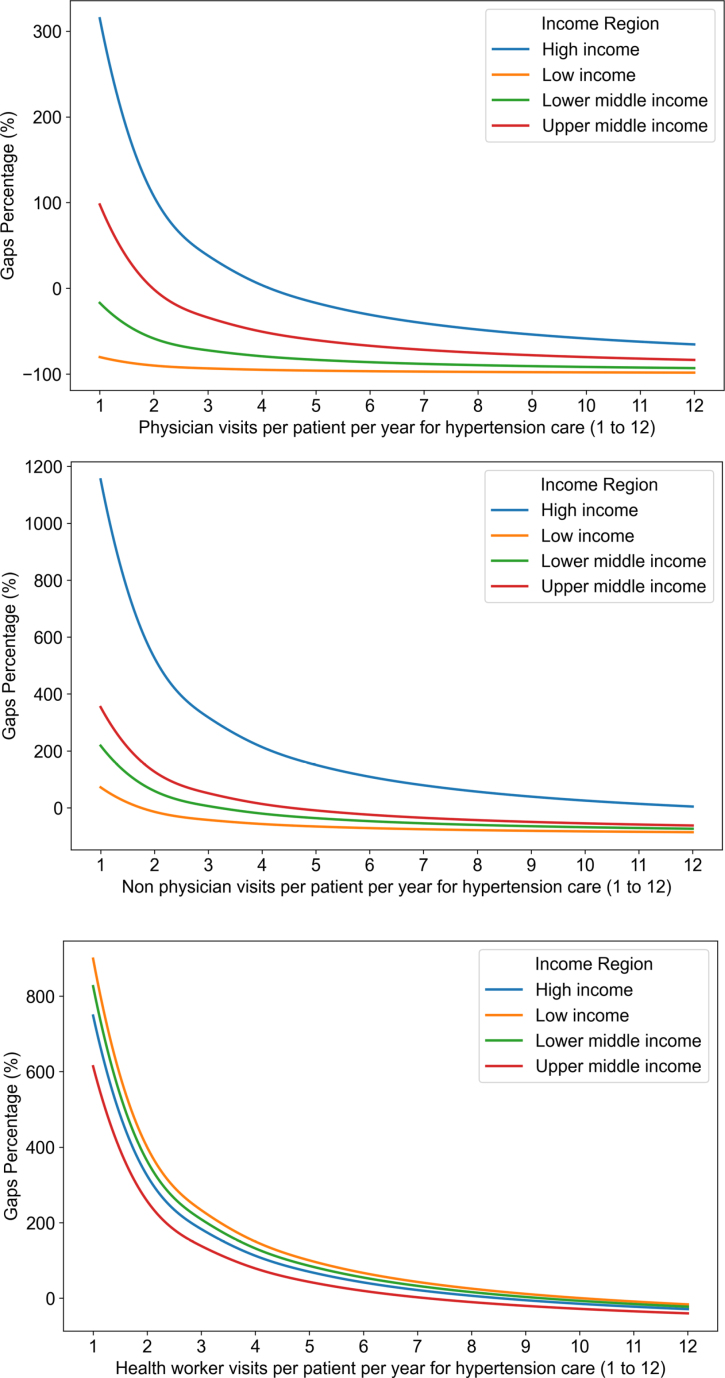
**Percentage of gap by number of visits per year, stratified by tier of income status (base scenario).** The percentage represents the cumulative gap between need and health system capacity for providing hypertension services for a particular region and scenario. These values may exceed 100%, indicating that the unmet need is more than double the available capacity. A negative value indicates that the demand for medical appointments by people with hypertension exceeds the available supply of physicians or nonphysician workers.

Overall, in the base high-demand scenario of 12 visits per patient per year, countries and territories lacked capacity for optimal hypertension care (Table; Figure 4A; Figure S1). These deficits persisted even after gradually reducing the number of visits from 12 to 6. HIC and UMIC showed smaller deficits after clinic visits were further reduced from 3 clinic visits to 1 visit.

**Table.**
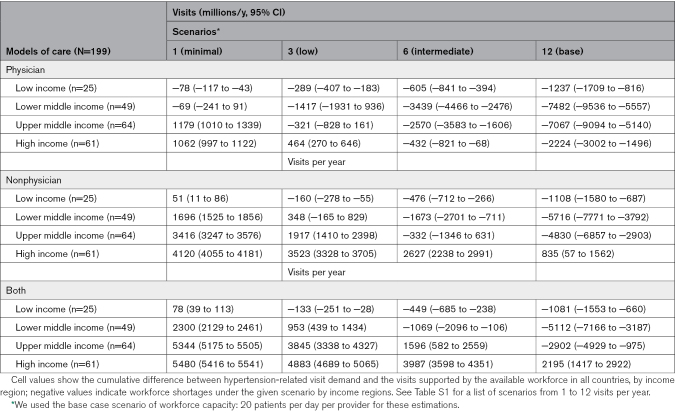
Cumulative Visit Gap (Millions Per Year, 95% CI) by Average Number of Visits Per Year and Income Regions

Under our base scenario of 12 visits per patient per year and 20 patients per provider, many countries in Africa, Asia, and South America showed a deficit in clinic visits (darkest red in Figure S1) regardless of the model of care (physician versus nonphysician or community health worker). In contrast, many countries in Europe and North America showed substantial surpluses in clinic visits under the same scenarios. These deficits persisted in Africa, Asia, and South America even under a low-capacity scenario when health workers examined 10 patients per day (darkest red in Figure S2B and S2C).

### Estimation of Clinical Visits Provided by Nonphysician Workers

Given the larger workforce sizes, nonphysician health workers provided higher health care visits compared with involving physicians alone. Most LICs and L-MIC showed a deficit in clinic visits when physicians alone were involved, but not when nonphysician health workers were involved in the workforce under our minimal scenario (Figures S2 and S3). For the remaining scenarios (ie, base, intermediate), involving nonphysician health workers did not completely remove these shortfalls, showing entrenched gaps in the workforce in LIC and LMIC.

### Sensitivity Analysis

We conducted a sensitivity analysis by using a high-capacity (20 patients per day) and low-capacity scenario (10 patients per day; Figure S1). All countries appeared to have clinical visits deficit when considering 12 visits per year per physician, as well as under 6 visits per year per physician. The largest deficit was found in India (3.6 billion visits) and Niue (0.005 million visits), respectively. Countries with the largest deficits and surpluses under 3 visits per year were India (0.7 billion visits) and China (0.14 billion visits), respectively; both India (0.7 billion visits) and China (2.0 billion visits) achieved significant surpluses when nonphysician health workers are involved in the workforce.

In addition, we explored what if both physicians and nonphysicians provide hypertension services under our base scenarios (20 patients per day), the extent of clinical visit deficits lessened, leaving 36 countries with surpluses in clinical visits (Figure S2). When both physicians and nonphysician in hypertension services, largest surpluses and deficits were found respectively in the United States (0.7 billion visits million) and India (2.2 billion).

## Discussion

We identified a substantial gap in health system capacity to meet demand for patient management of hypertension when reliant on a physician (12 visits per year per provider); with majority of countries failing to provide adequate hypertension care with high-patient throughput (20 patients per day) and low-patient throughput (10 patients per day). These deficits persisted even when considering 6 visits (intermediate), 3 visits (low), and 1 visit (minimal) to a physician per year in both high- and low-throughput models in LMICs, reaffirming findings from previous studies.^21,22^ Integrating nonphysician health workers into hypertension care reduced the size of deficits but did not eliminate them entirely. This highlights the critical shortage of health professionals affecting the provision of hypertension care in most LICs and L-MICs.

Our findings reinforce a well-documented challenge in global health—the persistent mismatch between healthcare provider capacity and patient needs, particularly in hypertension management.^21,22^ Although the disparities, especially in LIC and L-MIC regions, are expected, implementing sustainable and effective solutions remains a major challenge.^20^ Addressing these gaps requires multifaceted approaches, including workforce expansion, task-sharing, and integration of technology-enabled solutions.^4^ However, the success of such strategies depends on health system capacity, health financing, and political commitment, making implementation highly complex.^4^ Early investments in hypertension management could yield higher returns due to the gains realized by preventing complications associated with hypertension.^28^ For example, $1 invested in self-managed BP at home will provide $7.5 to 19.3 in return in terms of better health and productivity over 10 years.^28^ Despite recognizing that monthly visits are highly beneficial to patients, we found that the majority of countries do not have the capacity to meet demand based on the current availability of health professionals trained in providing hypertension services. Amid the global hypertension crisis, renewed focus is essential, particularly with substantial investment in building and deploying qualified human resources for hypertension services. Health systems in resource poor countries have arrived at a critical juncture to explore potential avenues to maximize hypertension services including exploring the feasibility of integration of tele-consultation and digital technologies.

Team-based care, involving coordinated efforts of healthcare professionals, such as doctors, nurses, and pharmacists, peer educators, community health workers, mothers’ groups), has been shown to significantly improve BP control rates, learning from deployment of similar workforce in HIV and maternal and child health programs.^18–20^ WHO’s HEARTS package is steadily expanding into an institutionalized model of care—now adopted in ≈6000 health centers across 40 countries, including those in Asia (eg, India, Bangladesh) and Africa (eg, Nigeria, Ethiopia), reaching 17.4 million people to date.^29,30^ HEARTS package implements several best practices to achieve optimal BP target among the population, through standardized clinical pathways, mass screening, early assessment of cardiovascular risk, simple treatment protocols involving single-pill combinations, and task-sharing among nonphysicians.^31^ A protocol-driven approach, where each team member follows standardized guidelines, ensures consistent monitoring, medication adherence, and patient education, leading to better hypertension management outcomes.^32,33^ Given the lack of trained workforce in low-income settings, skill transfer with nonphysician workforce can alleviate pressure on the already stretched health system.^34,35^ Therapeutic relationship, educational role, and clinical role that nurses perform are also likely to help patients manage their chronic disease better.^36^ Nonphysician health workers, including nurses, can be supported by additional training, simplified standard treatment protocols (algorithmic diagnosis and management), and consistent support by physicians by using face-to-face or digitally enhanced methods of coaching and mentoring.^34^ Such upskilling and support strategies should be customized according to the level of basic training these nonphysician health professionals have.^37^ This will help avoid subjecting community health workers to overburdening with too many responsibilities. Offering designated roles for the nonphysician health workers can also increase reach to the patients in the communities and lead to better clinical outcomes.^38^ Although there are several successful community-based interventions of chronic disease management using nonphysician health workforce,^38,39^ such approach may need to be contextualized to ensure that the multidisciplinary model of care works for each country’s context. Successful models should also be scaled up within similar contexts, so these initiatives go beyond pilots. Further research is needed to explore the feasibility, practicalities, including cost-effectiveness of such a provision.

There are 3 major issues that underpin the shortages of hypertension workforce in LMICs. First, there is a systematic draining of human resources from LMICs to HICs because of poor incentivization and insecurity.^40^ It is not uncommon to see a final year medical student and even a specialist doctor in LMICs preparing for an overseas career at any point after graduation.^41^ The years of investment in a health professional by an LMIC home country become readily available for HICs with minimal or no return on investment to LMICs.^42^ Second, the hypertension workforce is poorly defined, and education and training have not systematically focused on meeting the demand. There is widespread belief that hypertension services are dependent only on physicians. The nonphysician health workforce, such as hypertension nurses, remote area nurses with special hypertension training, allied health, and paramedical mid-level workforce, which serve the majority of the population, is unrecognized for its contribution. Although physicians are extremely important to ensure clinical service to people with hypertension, the other groups of health workforce are possibly the most feasible, yet largely untapped resources, within the health systems of LMICs. In HICs, such as the United States as well, reluctance to broaden the sharing of responsibilities with nonphysicians has crippled rural health services, inevitably affecting the care and follow-up for hypertension patients. Similarly, hypertension-related careers within the professional career ladder are also expected to be less attractive compared with other specialists due to factors such as work-life balance and professional outlook.^43,44^ Therefore, efforts are needed to improve the outlook of chronic disease management by providing better autonomy, well-defined career opportunities, incentives, and a support system for hypertension professionals.^15,22,45^

Besides LMICs, HICs like the United States, Canada, the United Kingdom, and France, which have near universal health coverage, variations in income, education, and geographic access influence healthcare capacity and frequency of clinical visits.^46,47^ These disparities highlight the need for targeted policies that address social determinants of health, ensuring equitable healthcare access within all economic strata. Our findings highlight the extent of disparities in health care capacity and underscore the importance of considering both systemic and socioeconomic factors when addressing healthcare capacity challenges globally.

The rapid expansion of technology, including smartphones and digital health solutions, presents significant opportunities for improving healthcare delivery, even in LMICs. Telemedicine, mobile health applications, and artificial intelligence–driven decision support systems have shown promise in enhancing accessibility, efficiency, and quality of care.^48^ However, the adoption of such solutions must consider contextual challenges, including digital literacy, infrastructure constraints, and socioeconomic disparities. Although technology offers a forward-looking approach to healthcare innovation, labor-intensive solutions remain crucial, particularly in settings where human engagement, trust, and community-based care play a central role. Therefore, a hybrid approach that integrates technology with workforce-driven strategies may be the most effective path forward.^4,48^

Our study has several limitations, and findings should be cautiously interpreted with further in-country analysis to support local policies and intervention, as noted in earlier studies.^21,22^ First, the World Bank data for physician and nonphysician density are not updated to recent years for the majority of countries. Although there is an increase in physician training annually owing to the increase in the number of medical schools and medical technology, the disproportionate distribution of physicians stifles the needs in remote and rural locations, including prominently in LMICs. Second, we included the age-standardized prevalence of hypertension conditions by NCD-RisC 2021 that excludes those <30 years.^3,49^ Third, our estimation that 10% of physicians working in hypertension are likely an overestimation of the health system capacity, narrowing the clinical visit deficit. Fourth, this is an aggregate, country-level analysis and therefore does not reflect the variation in health workforce capacity within the country. Generally, urban areas in LMICs have higher physician and nonphysician density compared with rural areas; therefore, we expect clinic visit deficit in urban areas to be narrow compared with rural areas. Fifth, another key limitation of this study is the ecological design that limits inclusion of health system factors, which influence access and provider capacity across different regions. While these factors significantly influence disparities in healthcare delivery, our study primarily focused on estimating provider capacity gaps. Future research should explore these system-level determinants to provide a more comprehensive understanding of healthcare accessibility and equity with regard to health services for hypertension management. Sixth, our analysis does not assess how these gaps have evolved over time. A temporal analysis could offer critical insights into the effectiveness of past health policies and the changing healthcare demands in different economic contexts. Another limitation of the study is reliance on point estimates (95% CI) from NCD-RisC, which may not have been updated to the current year, reflecting our exploratory intent to highlight indicative gaps consistent with previous studies.^21,22^ Although outside the scope of our study, future research could examine the impact of other evidence-based strategies, such as standardized protocols and single-pill combinations on BP control and demand on hypertension services globally.

### Perspectives

LMIC health systems continue to face challenges in meeting demand for hypertension management. Our analysis reveals a critical mismatch between available health workforce capacity and the service needs for hypertension care across 199 countries and provides a further imperative for the expansion of WHO’s HEARTS package, institutionalizing team-based hypertension services from the ground up. These findings underscore an urgent need to rethink hypertension service delivery, moving beyond traditional physician-centric approaches. Scalable solutions include investing in team-based care, expanding training for nonphysician providers, and leveraging digital tools to extend care access. As countries work toward universal health coverage and noncommunicable disease targets, reconfiguring the health workforce for team-based, decentralized care is not just pragmatic, it is essential.

### Conclusions

Our study identified significant disparities in health service capacity globally if hypertension management continues to rely solely on physicians. To address these gaps, countries should explore avenues to expand team-based care through expansion in clinical capacity and efficiency through the use of innovative technologies and engagement of nonphysician health workers, including the nursing workforce, mid-level community health workers, and other contextually suitable workforce, that can be trained in a fairly short duration. These can be facilitated by multidisciplinary models of care involving community-level nonphysician health workers with minimal training; such models of care are also relevant for HICs. It is up to the respective health systems to identify which models and technologies will fit best in their context. Future research should explore the feasibility and acceptability of team-based hypertension care strategies.

## ARTICLE INFORMATION

### Author Contributions

S.R. Mishra contributed to writing—review and editing, writing the original draft, methodology, formal analysis, data curation, and conceptualization. G. Satheesh and V. Khanal contributed to writing—review and editing, methodology, and conceptualization. B. Adhikari, D. Parker, D.S. Picone, N. Chapman, A. Schutte, and R.I. Lindley contributed to writing—review and editing, methodology, and conceptualization.

### Sources of Funding

The authors received no funding for this study. A.E. Schutte is funded by an Investigator Grant from the National Health and Medical Research Council of Australia (APP2017504).

### Disclosures

A.E. Schutte received speaker honoraria and consultancy fees from Servier, Abbott, Sanofi, AstraZeneca, Medtronic, Omron, Aktiia, and Sky Labs. The other authors report no conflicts.

## Supplementary Material


