# Supplementary material for: Bridging Hypertension Care Shortfalls Between Provider Capacity and Patient Needs: A Pooled Analysis of Data From 199 Countries and Territories
Source: Hypertension. 2025 Sep 26;82(11):1906–15. doi: 10.1161/HYPERTENSIONAHA.125.24783 (PMC12529994; doi:10.1161/HYPERTENSIONAHA.125.24783)
Supplement: Supplementary file 1 [file hyp-82-1906-s001.pdf]

SUPPLEMENTARY MATERIAL

Bridging hypertension care shortfalls between provider capacity and patient needs: a pooled analysis of data from 199 countries and territories

Short title: Bridging hypertension care shortfalls globally

Shiva Raj Mishra PhD[1,2,3], Gautam Satheesh PharmD [4], Vishnu Khanal PhD[3,5], Bipin Adhikari PhD[6], Daniel Parker PhD[7], Dean S. Picone PhD[4], Niamh Chapman PhD[4], Aletta E. Schutte PhD[8,9], Richard I Lindley MD[3],

Corresponding author:

COBIN Project, Nepal Development Society

Bagmati Province, Bharatpur, Metropolitan City-6, Chitwan, Nepal

Email: shivarajmishra@gmail.com

Supplementary Table S1. Total Gap in Visit (Millions/y, 95% CI) by Average Number of Visits per Year (Base Case Scenario of Physician and non-Physician Volume)

Supplementary Table S2. STROBE Statement—checklist of items that should be included in reports of observational studies.

Supplementary Figure S1. Difference between health system capacity and patient need under base scenarios (20 patients per day and 12 clinical visits per year). A. Non-physician B. Physician C. Both (team-based care)

Supplementary Figure S2. Difference between clinical capacity for hypertension care and patient need under a low-capacity scenario (10 patients per day and 12 visits per year). A. Non-physician B. Physician C. Both (team-based care)

Supplementary Figure S3. Percentage of countries with gap by number of visits per year, stratified by income groups (base scenario). Y-axis shows the percentage of countries where demand for medical appointments by people with hypertension exceeds the available supply of physicians or non-physician workers. A. Physician B. Non-Physician C. Both (team-based care).

Supplementary Table S1. Cumulative Gap in Visit (Millions/y, 95% CI) by Average Number of Visits per Year (Base Case Scenario of Physician and non-Physician Volume)

Income Region (N=199 Countries & territories)	Visits (millions/year)											
	1	2	3	4	5	6	7	8	9	10	11	12
Both (n=199)	13203 (12758 to 13619)	11375 (10487 to 12209)	9548 (8215 to 10798)	7720 (5943 to 9387)	5893 (3671 to 7976)	4065 (1400 to 6565)	2238 (-872 to 5155)	411 (-3144 to 3744)	-1417 (-5416 to 2333)	-3244 (-7688 to 922)	-5072 (-9959 to -489)	-6899 (-12231 to -1899)
Physician (n=199)	2093 (1648 to 2509)	265 (-623 to 1099)	-1562 (-2895 to -312)	-3390 (-5167 to -1723)	-5217 (-7439 to -3134)	-7045 (-9710 to -4545)	-8872 (-11982 to -5955)	-10699 (-14254 to -7366)	-12527 (-16526 to -8777)	-14354 (-18797 to -10188)	-16182 (-21069 to -11599)	-18009 (-23341 to -13009)
Non-Physician (n=199)	9282 (8838 to 9699)	7455 (6566 to 8288)	5628 (4295 to 6878)	3800 (2023 to 5467)	1973 (-249 to 4056)	145 (-2521 to 2645)	-1682 (-4792 to 1234)	-3510 (-7064 to -176)	-5337 (-9336 to -1587)	-7165 (-11608 to -2998)	-8992 (-13880 to -4409)	-10820 (-16151 to -5820)
**Physician**												
Low income (n=25)	-78 (-117 to -43)	-183 (-262 to -113)	-289 (-407 to -183)	-394 (-551 to -254)	-499 (-696 to -324)	-605 (-841 to -394)	-710 (-985 to -465)	-815 (-1130 to -535)	-921 (-1275 to -605)	-1026 (-1419 to -675)	-1131 (-1564 to -746)	-1237 (-1709 to -816)
Lower middle income (n=49)	-69 (-241 to 91)	-743 (-1086 to -422)	-1417 (-1931 to -936)	-2091 (-2776 to -1449)	-2765 (-3621 to -1963)	-3439 (-4466 to -2476)	-4112 (-5311 to -2990)	-4786 (-6156 to -3503)	-5460 (-7001 to -4016)	-6134 (-7846 to -4530)	-6808 (-8691 to -5043)	-7482 (-9536 to -5557)
Upper middle income (n=64)	1179 (1010 to 1339)	429 (91 to 750)	-321 (-828 to 161)	-1070 (-1746 to -428)	-1820 (-2665 to -1017)	-2570 (-3583 to -1606)	-3319 (-4502 to -2195)	-4069 (-5420 to -2784)	-4818 (-6339 to -3373)	-5568 (-7257 to -3962)	-6318 (-8176 to -4551)	-7067 (-9094 to -5140)
High income (n=61)	1062 (997 to 1122)	763 (633 to 884)	464 (270 to 646)	166 (-94 to 408)	-133 (-457 to 170)	-432 (-821 to -68)	-730 (-1184 to -306)	-1029 (-1548 to -544)	-1328 (-1911 to -782)	-1626 (-2275 to -1020)	-1925 (-2638 to -1258)	-2224 (-3002 to -1496)
**Non-Physician**												
Low income (n=25)	51 (11 to 86)	-55 (-133 to 15)	-160 (-278 to -55)	-265 (-423 to -125)	-371 (-568 to -196)	-476 (-712 to -266)	-582 (-857 to -336)	-687 (-1002 to -406)	-792 (-1146 to -477)	-898 (-1291 to -547)	-1003 (-1436 to -617)	-1108 (-1580 to -687)

Lower middle income (n=49)	1696 (1525 to 1856)	1022 (680 to 1343)	348 (-165 to 829)	-326 (-1010 to 316)	-999 (-1855 to -197)	-1673 (-2701 to -711)	-2347 (-3546 to -1224)	-3021 (-4391 to -1738)	-3695 (-5236 to -2251)	-4369 (-6081 to -2765)	-5043 (-6926 to -3278)	-5716 (-7771 to -3792)
Upper middle income (n=64)	3416 (3247 to 3576)	2666 (2328 to 2987)	1917 (1410 to 2398)	1167 (491 to 1809)	417 (-427 to 1220)	-332 (-1346 to 631)	-1082 (-2264 to 42)	-1831 (-3183 to -547)	-2581 (-4101 to -1136)	-3331 (-5020 to -1725)	-4080 (-5939 to -2314)	-4830 (-6857 to -2903)
High income (n=61)	4120 (4055 to 4181)	3822 (3692 to 3943)	3523 (3328 to 3705)	3224 (2965 to 3467)	2926 (2601 to 3229)	2627 (2238 to 2991)	2328 (1874 to 2752)	2030 (1511 to 2514)	1731 (1147 to 2276)	1432 (784 to 2038)	1134 (420 to 1800)	835 (57 to 1562)
**Both (team-based care)**												
Low income (n=25)	78 (39 to 113)	-27 (-106 to 43)	-133 (-251 to -28)	-238 (-395 to -98)	-343 (-540 to -168)	-449 (-685 to -238)	-554 (-829 to -309)	-659 (-974 to -379)	-765 (-1119 to -449)	-870 (-1263 to -519)	-975 (-1408 to -590)	-1081 (-1553 to -660)
Lower middle income (n=49)	2300 (2129 to 2461)	1627 (1284 to 1947)	953 (439 to 1434)	279 (-406 to 920)	-395 (-1251 to 407)	-1069 (-2096 to -106)	-1743 (-2941 to -620)	-2416 (-3786 to -1133)	-3090 (-4631 to -1647)	-3764 (-5476 to -2160)	-4438 (-6321 to -2674)	-5112 (-7166 to -3187)
Upper middle income (n=64)	5344 (5175 to 5505)	4594 (4257 to 4916)	3845 (3338 to 4327)	3095 (2419 to 3738)	2346 (1501 to 3148)	1596 (582 to 2559)	846 (-336 to 1970)	97 (-1255 to 1381)	-653 (-2173 to 792)	-1403 (-3092 to 203)	-2152 (-4010 to -386)	-2902 (-4929 to -975)
High income (n=61)	5480 (5416 to 5541)	5182 (5052 to 5303)	4883 (4689 to 5065)	4584 (4325 to 4827)	4286 (3962 to 4589)	3987 (3598 to 4351)	3688 (3235 to 4113)	3390 (2871 to 3875)	3091 (2508 to 3637)	2792 (2144 to 3398)	2494 (1781 to 3160)	2195 (1417 to 2922)

*Included countries where physician and non-Physician density (per 10,000 population) could be available. Cell values show the cumulative difference between hypertension-related visit demand and the visits supported by available workforce in all countries, by income region; negative values indicate workforce shortages under the given scenario by income regions.

Supplementary Table S2. STROBE Statement—checklist of items that should be included in reports of observational studies

	Item No	Recommendation	Page No
**Title and abstract**	1	(*a*) Indicate the study's design with a commonly used term in the title or the abstract	Page 1
		(*b*) Provide in the abstract an informative and balanced summary of what was done and what was found	Page 2
**Introduction**			
Background/rationale	2	Explain the scientific background and rationale for the investigation being reported	Page 3
Objectives	3	State specific objectives, including any prespecified hypotheses	Page 3
**Methods**			
Study design	4	Present key elements of study design early in the paper	Page 4-5

Setting	5	Describe the setting, locations, and relevant dates, including periods of recruitment, exposure, follow-up, and data collection	Page 4-5
Participants	6	*Cross-sectional study*—Give the eligibility criteria, and the sources and methods of selection of participants	Page 4
Variables	7	Clearly define all outcomes, exposures, predictors, potential confounders, and effect modifiers. Give diagnostic criteria, if applicable	Page 5
Data sources/ measurement	8*	For each variable of interest, give sources of data and details of methods of assessment (measurement). Describe comparability of assessment methods if there is more than one group	Page 5
Bias	9	Describe any efforts to address potential sources of bias	Page 5-6, *Estimation method for health system capacity and patient's need.*
Study size	10	Explain how the study size was arrived at	Page 5-6
Quantitative variables	11	Explain how quantitative variables were handled in the analyses. If applicable, describe which groupings were chosen and why	Page 5-6, *Estimation method for health system capacity and patient's need.*
Statistical methods	12	(*a*) Describe all statistical methods, including those used to control for confounding	Page 5-6, *Estimation method for health system capacity and patient's need.*
		(*b*) Describe any methods used to examine subgroups and interactions	
		(*c*) Explain how missing data were addressed	
		Cross-sectional study—If applicable, describe analytical methods taking account of sampling strategy	
		(*e*) Describe any sensitivity analyses	Page 6.
**Results**			
Participants	13*	(a) Report numbers of individuals at each stage of study—eg numbers potentially eligible, examined for eligibility, confirmed eligible, included in the study, completing follow-up, and analysed	Page 5, Prevalence of hypertension and health system's capacity
		(b) Give reasons for non-participation at each stage	NA
		(c) Consider use of a flow diagram	NA
Descriptive data	14*	(a) Give characteristics of study participants (eg demographic, clinical, social) and information on exposures and potential confounders	Page 5, Prevalence of hypertension and health system's capacity
		(b) Indicate number of participants with missing data for each variable of interest	NA
Outcome data	15*	*Cross-sectional study*—Report numbers of outcome events or summary measures	Page 5, Prevalence of hypertension and health system's capacity
Main results	16	(*a*) Give unadjusted estimates and, if applicable, confounder-adjusted estimates and their precision (eg, 95% confidence interval). Make clear which confounders were adjusted for and why they were included	Page 5-6
		(*b*) Report category boundaries when continuous variables were categorized	NA
		(*c*) If relevant, consider translating estimates of relative risk into absolute risk for a meaningful time period	NA

Other analyses	17	Report other analyses done—eg analyses of subgroups and interactions, and sensitivity analyses	Page 6
**Discussion**			
Key results	18	Summarise key results with reference to study objectives	Page 6
Limitations	19	Discuss limitations of the study, taking into account sources of potential bias or imprecision. Discuss both direction and magnitude of any potential bias	Page8
Interpretation	20	Give a cautious overall interpretation of results considering objectives, limitations, multiplicity of analyses, results from similar studies, and other relevant evidence	Page 6-7
Generalisability	21	Discuss the generalisability (external validity) of the study results	Page 6-7
**Other information**			
Funding	22	Give the source of funding and the role of the funders for the present study and, if applicable, for the original study on which the present article is based	Page 10

*Give information separately for cases and controls in case-control studies and, if applicable, for exposed and unexposed groups in cohort and cross-sectional studies.

Note: An Explanation and Elaboration article discusses each checklist item and gives methodological background and published examples of transparent reporting. The STROBE checklist is best used in conjunction with this article (freely available on the Web sites of PLoS Medicine at http://www.plosmedicine.org/, Annals of Internal Medicine at http://www.annals.org/, and Epidemiology at http://www.epidem.com/). Information on the STROBE Initiative is available at www.strobe-statement.org.

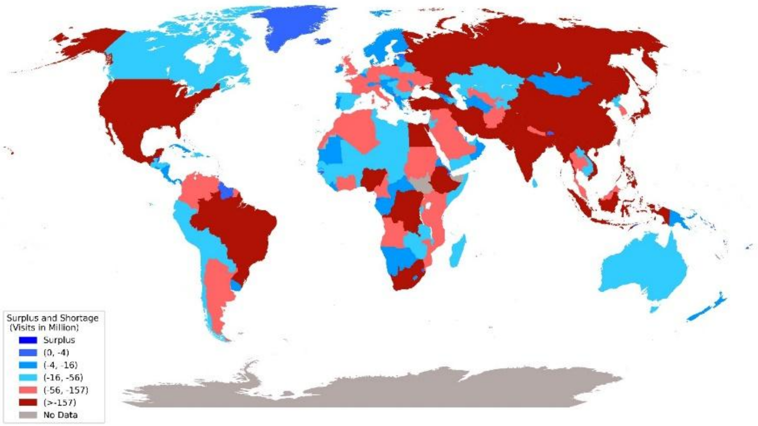

(A) Physician

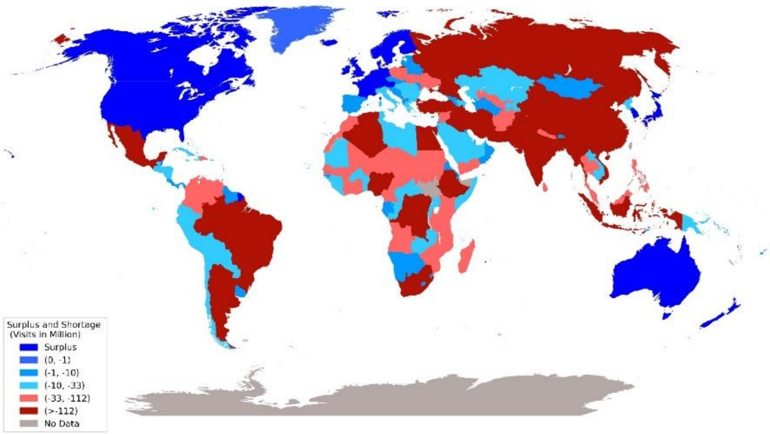

(B) Non-physician

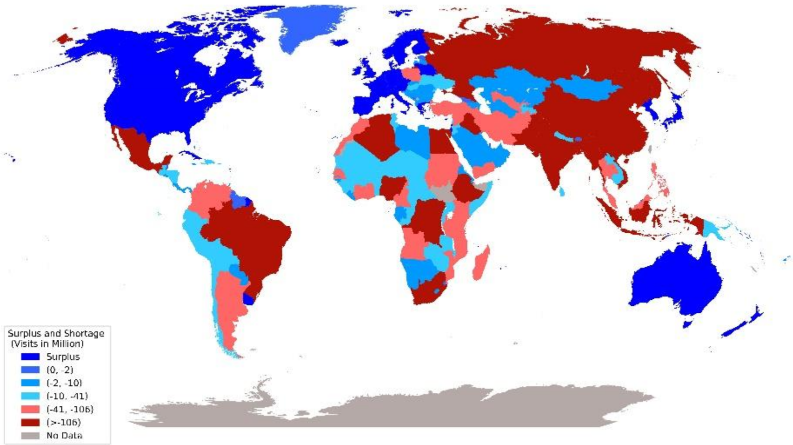

(C) Both (team-based care)

Supplementary Figure S1. Difference between health system capacity and patient need under base scenarios (20 patients per day and 12 clinical visits per year). A. Physician B. Non-Physician C. Both (team-based care)

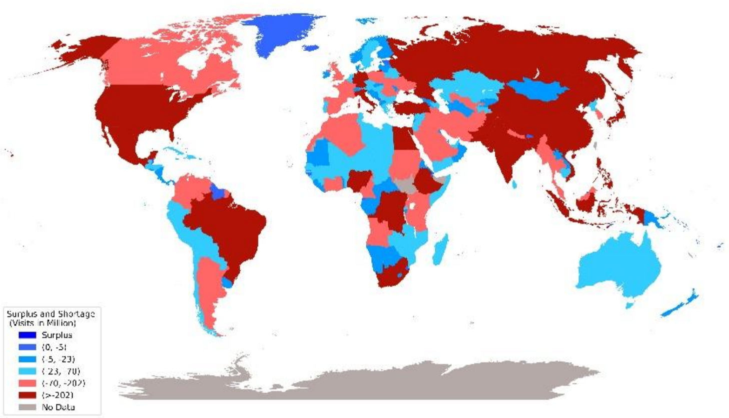

A Physician

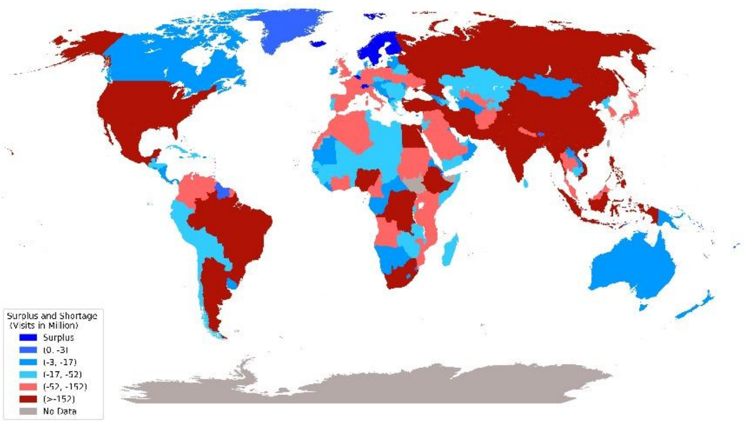

B Non-physician

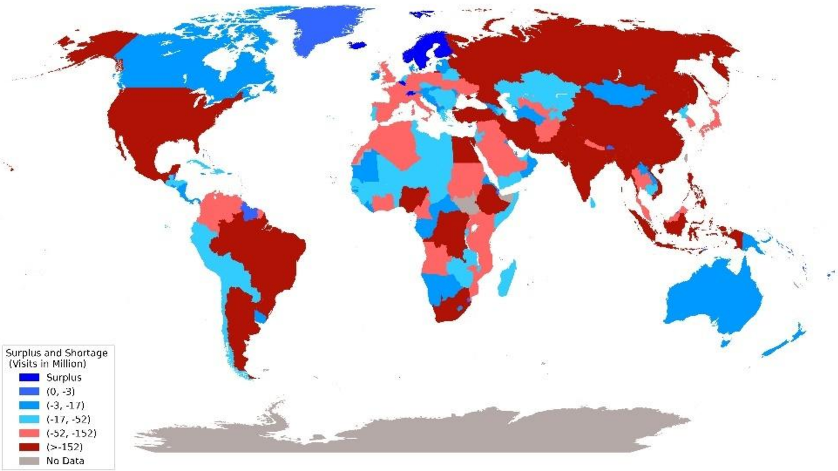

C Both (team-based care)

Supplementary Figure S2. Difference between clinical capacity for hypertension care and patient need under a low-capacity scenario (10 patients per day and 12 visits per year). A. Physician B. Non-Physician C. Both (team-based care)

A

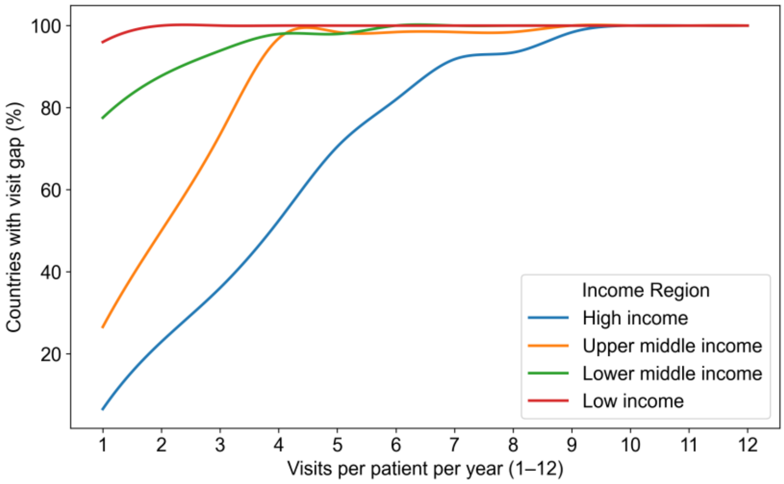

A Physician

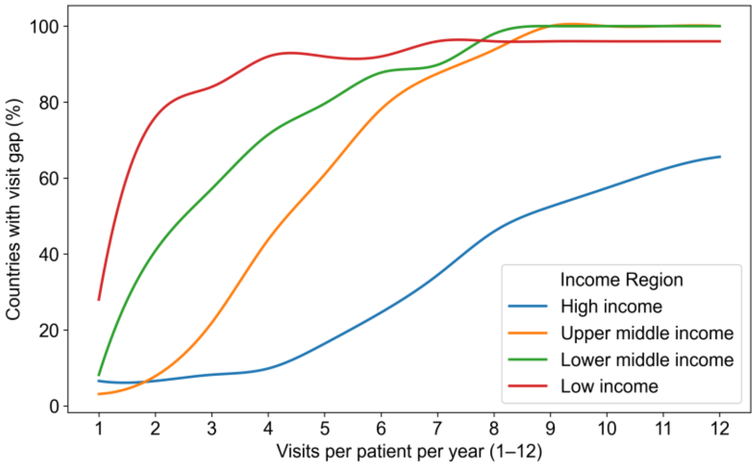

A Non-Physician

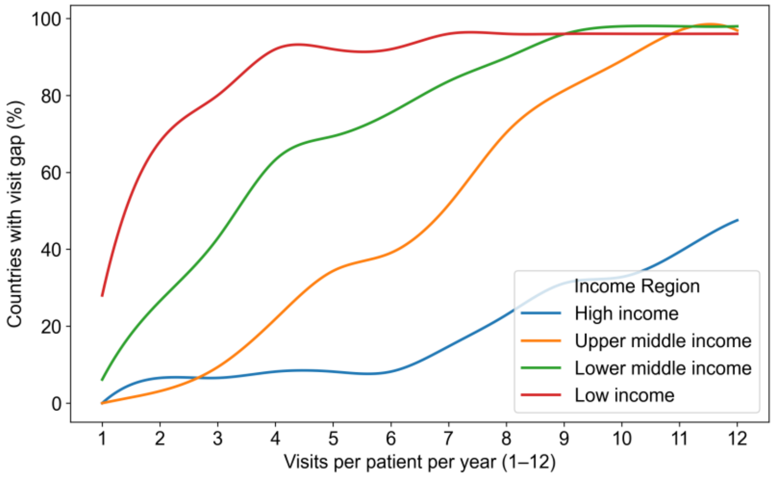

C Both (team-based care)

Supplementary Figure S3. Percentage of countries with gap by number of visits per year, stratified by income groups (base scenario). Y-axis shows the percentage of countries where demand for medical appointments by people with hypertension exceeds the available supply of physicians or non-physician workers. A. Non-physician B. Physician C. Both (team-based care).
